# Understanding kuru: the contribution of anthropology and medicine

**DOI:** 10.1098/rstb.2008.0072

**Published:** 2008-11-27

**Authors:** Shirley Lindenbaum

**Affiliations:** Graduate School and University Center, City University of New York365 Fifth Avenue, New York, NY 10016-4309, USA

**Keywords:** kuru, anthropology, endocannibalism

## Abstract

To understand kuru and solve the problems of its cause and transmission required the integration of knowledge from both anthropological and medical research. Anthropological studies elucidated the origin and spread of kuru, the local mortuary practices of endocannibalism, the social effects of kuru, the life of women and child-rearing practices, the kinship system of the Fore and their willingness to incorporate outsiders into it, the myths, folklore and history of the Fore and their neighbours, sorcery as a powerful social phenomenon and way of explaining the causation of disease, and concepts of the treatment of disease. Many scientists from different disciplines, government officers and others have contributed to this chapter of medical history but it is the Fore people who have contributed the most, through their suffering, their cooperative and reliable witness to kuru, and their participation, in various ways, in the research process itself.

## 1. Introduction

Scientific investigation of kuru began in 1957, and by 1961 a genetic explanation was most favoured among medical investigators. With a grant from the Rockefeller Foundation provided by Henry Bennett at the University of Adelaide, Robert Glasse and I began our anthropological study of kuru in July 1961. Bennett, one of the first to propose the genetic hypothesis (Bennett *et al.* 1959), asked that we study Fore kinship and, in particular, gather the genealogical data he thought would confirm his theory. This essay provides an account of our research from 1961 to 1963, with some observations based on the fieldwork I carried out between 1970 and 1999.

Anthropology is both a natural science and a humanistic discipline, mediating between human biology and ecology on one hand and the study of human understanding on the other. By entering as fully as possible into the everyday life of others, anthropologists are, of necessity, both outside observers and participants in the internal dialogues of the people with whom we live (Wolf 1974, p. 13). Taking seriously Bennett's charge to investigate kinship, a key topic for anthropologists, we learned Fore kin terms, drew genealogical charts, observed kinship in action and began to understand something of Fore domestic and political relations (Glasse & Lindenbaum 1969, 1980). We also recorded Fore beliefs about kuru, their accounts of the history of the epidemic and of mortuary practices and observed the treatment of kuru victims by local healers. This provided us with a view of the epidemic at odds with the genetic hypothesis and allowed us to suggest an alternative reading, confirmed later by medical investigators.

## 2. Fore political and domestic relations

Bennett had asked that we document Fore ‘pedigrees’, a clue to the problem that lay ahead. It soon became apparent that many of the kuru victims were not closely related biologically, but were kin in a non-biological sense. Our genealogical investigations led us to document the wider social structures within which kinship was situated, providing the context for interpreting the person-to-person connections inscribed on our kinship charts.

The Fore named large regional clusters to which they believed they belonged (Ibusa, Atigina, Pamusa), but these district associations were misty entities with small differences in dialect and custom. The more meaningful units were smaller political entities, which we called parishes, consisting of one or several adjacent hamlets, the members having corporate interest in a defined territory and sharing a ‘spirit place’ or sacred grove. Ideally, these units joined for defence and settled internal quarrels peaceably. In the South Fore population of approximately 7000 in the early 1960s, 39 such units ranged in size from 41 to 525, with a mean of 180. The smallest parish subdivision was the Fore *lounei*, the line, a group of people who thought of themselves as descendants of a named patrilineal ancestor, who usually resided together and were exogamous, with a preference for men to marry their mother's brother's daughters. Allied lines were subject to a single incest taboo. They joined also in assembling bride price and in giving their own deceased kin to be consumed by the ‘line’ of their mothers' brothers, reinforcing the customary exchange of other items of value (pigs, shells, bodily substance) between kin related by kinship and marriage.

Unity and harmony within the political units, however, was tenuous. Immigrant lines formed enclaves and enjoyed dual rights as long as they continued to visit and maintain an interest in their original group. The acceptance of immigrant groups solved a problem facing colonizing populations, especially the Fore at that time—the shortage of marriageable women. The burden fell on newcomers whose incorporation into the group depended on demonstrated loyalty and observance of their new kinship obligations. These political units were said to possess ‘one blood’ and to stem from a common ancestor, conveying the idea of unity of those who reside and act together, and who also share the bodily substance of those who eat food grown on their land. In time, individuals who demonstrated continued commitment to their adopted group came to occupy the kinship status of ‘brother’ or ‘sister’ that these titles signified. Reference to common ancestors and common substance defined kinship status and provided a moral guide for living, but were not reliable statements of genetic relationships.

Fore genealogies were short, no more than five generations deep, two above and two below the young or middle-aged adult who provided the information. Instead of depth, the Fore relied on lateral expansions of relatedness. They readily permitted adoption, finding substitute parents in the father's line for children orphaned by the death of their mothers by kuru and, in the past, the loss of their fathers by war injuries. Newly married couples were ‘lent’ infants by close kin, and adoptions took place also among less closely related people, gifts of fertility and labour eliciting a reciprocal exchange of wealth that underwrote the bond between the two parties (see Strathern 1988).

A more frequent kinship elaboration occurred with the creation of *kagisa* kin (from *kagine*, the time of the mid-day sun). Individuals with no known consanguineal relationship exchanged food and wealth in a formal meal during the *kagine*, when the sun was directly overhead. Sanctioned by the sun, a cosmic being, and sealed with the consumption of food grown on home territory, this tie also established the kinship of common substance. *Kagisa* kinship was important for creating mothers' brothers in a society with a preference for men to marry a mother's brother's daughter, and for creating sisters who could provide brothers with a nearby source of food and affection, and a portion of bride price when the *kagisa* sister married. Fore genealogies were thus social documents that gave legitimacy to the claims and obligations of kinship.

One additional relationship concerns *wagoli* (‘base’ or ‘root’ men), war allies and trade partners whose territories were in the past considered places of refuge. *Wagoli* received a portion of their partner's death payment. Each of them provided the other with elaborate hospitality, sisters of the host *wagoli* became *kagisa* sisters of the visitor and the host's children his ‘sons’ and ‘daughters’. Some *wagoli* relationships were inherited from their parents, some they established themselves.

Over time, commitment to group defence and the sharing of resources tended to outweigh distant origin. The genealogies indicated that the Fore had ‘made invisible’ the origin of several adult men who were said to have belonged to a population that once lived south of Purosa, and who spoke Pawaian, a language not related to the East New Guinea Language Stock. Following a period of apparently harmonious interaction between the Fore and the Pawaians, the relationship had soured. The Fore burned down Pawaian houses and shot most of those who attempted to run away. The survivors, now adults, ‘became Fore’, and although we were told who they were, we were advised not to identify them. Our genealogical research thus indicated that the Fore definition of relatedness included people said to possess ‘one blood’, many of whom had acquired the status of close kinship by social means (Lindenbaum in press). In a number of ways our research had begun to indicate that a simple hereditary explanation for kuru seemed hard to justify.

Fore kinship can best be described as formed by webs of attachment based on lateral extension rather than vertical depth, on optional bonding not simply biological ascription. This is a form of social organization suited to a mode of agricultural subsistence in which fields are frequently relocated, the population is relatively mobile, and groups fragment and recombine in new alignments. Most adult men in 1961–1962 reported residing in different places at birth, initiation, marriage and fatherhood.

Much has changed in the South Fore since the early 1960s. The Fore no longer consume deceased kin and kuru is thus no longer transmitted. With the waning of the epidemic, and public health services that have reduced infant mortality, the population has increased rapidly. The shortage of women is no longer a concern, most people have abandoned pig keeping, and wage labour and markets have supplanted the indigenous trade networks that provided access to resources. With the suppression of warfare (and the creation of refugees), as well as the production of coffee as a cash crop (tying people to their plantations), the Fore population is now less mobile. A kinship system fashioned to meet the social conditions that existed 50 years ago may no longer be entirely relevant. It is probable that some features that once characterized Fore kinship, such as the ready incorporation of immigrants, practices of adoption and the widespread creation of *kagisa* kin, may not be well suited to the current needs. ‘Wantoks’ (friends) seem to be a supplement to *wagoli.* Contemporary genealogies may resemble more closely the pedigrees that Bennett had in mind.

## 3. The recent appearance of kuru

The data we gathered in 1962 indicated that kuru had spread slowly through Fore villages within living memory, and that its progress through Fore territory followed a specific, traceable route (Glasse 1962). This finding was at odds with a purely genetic model which implied that kuru must have been of remote evolutionary origin, and that it ought to have been in epidemiological equilibrium. As John Mathews observed later (Mathews 1971, pp. 13–14), kuru was too common and too fatal to be a purely genetic disorder unless the hypothetical kuru gene was maintained at high frequency by a mechanism of balanced polymorphism, for which there was no evidence.

The Fore reported that kuru had entered Fore territory from Uwami, a Keiagana village to their northwest *ca* 1920, and that the disease had travelled down the eastern border and then swung westward into central South Fore. From here, it turned again to the north and continued also to move south. Its appearance in the extreme south was thus relatively late, and many people gave persuasive accounts of their first encounter with the disease. We spent some weeks walking along the described route, visiting hamlets and collecting historical accounts. These stories placed the arrival of kuru at Kamira, adjacent to Wanitabe, in the late 1920s, and at Wanitabe (where we were based) by *ca* 1930. The first cases at Purosa, six miles south of Wanitabe, were also said to have occurred in the early 1930s. From scores of accounts, a broad chronology emerged of the arrival of kuru in some southwestern and southeastern areas as late as the 1940s (see the map (Lindenbaum 1979, p. 18) adapted from Mathews (1971)). Kuru was said to have appeared first among young women, with a subsequent shift to children of both sexes and adult men, an account that matched early epidemiological reports. Zigas & Gajdusek's (1959) assessment had noted the high incidence of kuru in certain families and hamlets, its localization to the Fore and adjacent people with whom they intermarried and its predilection for children and adult women.

Our genealogical records, which also recorded causes of death, confirmed Fore assertions that the disease was not of great historical depth. Deaths from kuru clustered in generations of young people and their parents, but were extremely rare in the next ascending generation. Moreover, the Fore could name for us and for later investigators those who had died of kuru. They could also name those who had participated in the consumption of deceased persons, demonstrating the link between the disease and cannibalism. As a result, a coherent account could be made for the appearance of the disease some 4–20 years after the ingestion of poorly cooked tissues containing the transmissible agent (Mathews *et al.* 1968).

In addition to the evidence we provided that kuru was a recent phenomenon, we thought also that the South Fore had adopted mortuary cannibalism in recent times, which we estimated to be roughly a decade before the appearance of kuru in the north, i.e. *ca* 1890 or 1900. In the 1960s, many Fore said that the practice had arrived from the Kamano in the north, and from the Markham Valley and the Agarabi people to the northwest. Jerome Whitfield, an anthropologist currently working in the South Fore, has evidence that the custom may be of greater historical depth, dating back six generations. Cannibalism, however, remains a significant factor in the transmission of the disease.

## 4. Kuru and cannibalism

During 1961 and 1962 we gathered detailed information about the practice of cannibalism and continued to do so in 1963. On 10 April 1963, we sent a report of our fieldwork (Glasse & Glasse 1963) to John Gunther, the Director of Public Health, and the source of our grant money for the second year of research. The report notes that we were continuing to gather information on seven topics begun earlier: the origin and spread of kuru; cannibalism and kuru; the social effects of kuru; women's life and child-rearing practices; basic kinship studies; myths, folklore and history; and concepts of disease treatment. On cannibalism and kuru we noted that ‘Extensive data has been collected on the possibility of an association between cannibal practices and the spread of kuru. As these practices vary considerably in the kuru region and in adjacent areas, an attempt will be made to relate these findings to variations in kuru prevalence. The data collected from the borders of the kuru region are of particular interest, and these will be discussed in relation to the spread of kuru’. We subsequently published papers on these topics (Glasse 1963, 1964, 1967; Lindenbaum & Glasse 1969; Lindenbaum 1971, 1976; Glasse & Lindenbaum 1976).

Our thoughts about the relationship between kuru and cannibalism rested heavily on data we had collected concerning Fore rules about the consumption of human flesh, which seemed to fit the epidemiological evidence available to us at that time. Although it was no longer present in the 1960s, having been suppressed under pressure from the government and missions, the Fore spoke openly about the recent customary practices of consuming the dead. The first government patrols in the late 1940s had also reported cannibalism to be customary throughout the region. Beyond the Fore, however, it was customary to consume enemies (exocannibalism), not deceased kin (endocannibalism), a pattern of behaviour with consequences for the transmission and geographical boundaries of kuru.

The anthropologists Ronald and Catherine Berndt, who carried out research among the North Fore and neighbouring populations from 1951 to 1953, said that cannibalism had ceased in the north by the 1950s, but was still practised surreptitiously in the south (Berndt 1962). The South Fore confirmed that they had indeed continued to hide and eat deceased kin until the mid-1950s, when a government road was built to provide access from Okapa in the north to the southern hamlets at Purosa. Thus, in the South Fore, the area with the highest incidence of kuru in the 1960s, cannibalism had continued longer than in the north.

**Figure 1:**
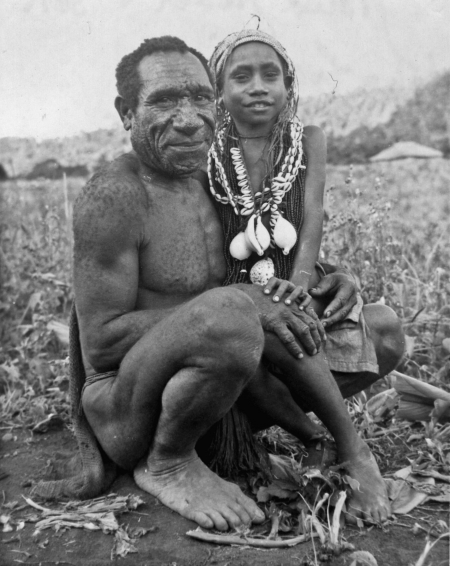
Fore initiate, 1961. Accompanied by his father he visits relatives to exhibit his new status.

All body parts were eaten, except the gall bladder that was considered too bitter. Not all bodies were eaten. The Fore did not eat those who died of dysentery, leprosy and possibly yaws, but kuru victims were viewed favourably. Most significantly, not all Fore were cannibals. Cannibalism among adult men in the North Fore occurred more frequently than it did in the south; in the south, men rarely ate human flesh, and those who did said they avoided eating the bodies of women. Small children residing in houses with their mothers ate what their mothers gave them. Initiated youths who moved to the communal men's house approximately at age 10 left behind the world of immaturity, femininity and cannibalism (figure 1). Consumption of human flesh was thus largely limited to adult women, children of both sexes and a few adult men, a pattern that matched the epidemiology of kuru in the early 1960s.

Our anthropological findings received little, often sceptical attention, until the anthropological and medical stories came together in 1966, when chimpanzees injected with brain material from victims of the disease exhibited a clinical syndrome akin to kuru (Gajdusek *et al.* 1966). This gave credence to the cannibalism hypothesis, as did the fact that following a change in this mortuary custom, kuru disappeared among children, while the age of those afflicted with the disease also rose (Alpers 1968).

Although we had no satisfactory medical model for explaining how the disease might be transmitted, we often spoke about kuru and cannibalism to those who visited us in the field, including Richard Hornabrook, who visited in May 1963, and later that month Norma McArthur, Jon Hancock, Michael Alpers and MacFarlane Burnet. Burnet later recorded an account of his visit, his initial reservations, and a subsequent shift to at least an open mind on the matter; this matter is discussed in more detail in Glasse & Lindenbaum (1992). For many people, cannibalism is a topic that elicits feelings of unease. During the 1970s, it became fashionable even within some anthropological circles to assert that institutionalized cannibalism never existed. In response, many anthropologists reevaluated data they had been reluctant to publish, and more nuanced studies were presented based on the research in Papua New Guinea, China and Africa (Lindenbaum 2004).

## 5. The social impact of kuru

**Figure 2:**
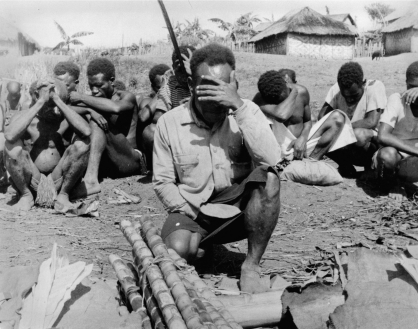
Bride price presentation, 1962. Men pray that the bride will survive and produce children.

In addition to investigating the history and transmission of kuru, we also examined the Fore experience and response to the epidemic, and how they explained it to themselves. Between 1957 and 1977, some 2500 people died of kuru, most of them adult Fore women. The pronounced sexual bias in kuru mortality was one of its most deranging aspects. In 1962, a sample of 125 Wanitabe males over the age of 21 showed that 63 had no living wives and 10 had never married. Women often died of kuru shortly after giving birth to a child. The motherless nuclear family was a common domestic unit. Many men were thus forced to perform the roles of both mother and father. Some assistance was provided by sisters and brothers' wives, and small daughters often worked long hours in the gardens, but men took on many domestic activities once considered as the woman's sphere. In addition to clearing and fencing garden sites, well-recognized male labours, men now began to dig the ground, plant crops, weed and harvest, becoming progressively involved in women's tasks as their wives' capacities began to wane. Sometimes they cooked food and fed the children. Bride price was now withheld until the bride had survived long enough to produce a child (figure 2). Marriage speeches during the distribution of bride price often included directions for the distribution of the bride's death payment.

Faced with a demographic emergency, the dimensions of which they grasped clearly, the South Fore had recourse to a series of desperate remedies (Lindenbaum 1979, pp. 89–116). During 1961 and 1962 the Fore expended much time, material wealth and emotional energy in an attempt to locate the sorcerers they believed to be responsible for the calamity. They also consulted a variety of curers in distant locations, taking ambulant victims of the disease on healing pilgrimages, the most spectacular of which took place among the neighbouring Gimi people. Between April and August 1961, more than 70 patients consulted a Gimi curer whose therapy consisted of bloodletting, the ingestion of medicinal barks and leaves, and the identification of the location where the guilty sorcerer might be found. Back at home, the sick women sometimes revealed the identity of their aggressor, said to have come to them in a dream. With the women present, men also conducted divination tests to reveal the sorcerers' identities, which often led to new tensions when the tests suggested that the sorcerers might be close neighbours and relatives. To the often expressed fear of extinction from the loss of women's reproductive power was now added a fear of internal disruption so great that their future was in danger.

This was the setting in which the Fore began to hold public meetings to denounce the acts of sorcery, speak about past animosities and reveal the concealed thoughts that they said gave rise to acts of aggression. In one community after another, from the beginning of November 1962 to the middle of March 1963, groups gathered to discuss the emergency. Local leaders proposed that they would tell the kiap (the colonial government officer) that men were killing their women. They would also ask him to take all the men away to a ‘place nothing’, leaving only women and children behind. After some time in this remote place they would then return and see if kuru had finished or not.

The threat of banishment derived its force from the colonial regime's access to armed police, and the ability to jail those who disobeyed the new laws. Government patrols, though infrequent, caused a ripple of anxiety as the official party, including the police, camped for several days in selected communities, where they carried out a census, identified people with leprosy to be sent for treatment at a distant government hospital and adjudicated disputes that local groups had been unable to resolve.

The central issue being debated at the meetings concerned whether kuru was the result of sorcery or a form of ‘sickness’. The knowledge that the kiap and kuru investigators considered kuru to be a sickness provided the counterpoint for all public discussions. Adoption of the word sickness did not mean that the Fore shared western medical concepts of biologically caused ailments based on germ theory. In this context sickness referred to illnesses not caused by the aggressive acts of men. (Non-sorcery-caused ailments were often said to be caused by encroachments against nature spirits, ghosts of the recently dead or angry neighbours, all of whom could be given compensation payments in order to find relief.) Wrestling with conflicting explanations, the Fore drew on their understanding of the nature of social relationships, of losses that required retaliation in a society based on reciprocal exchange, where wealth is transacted not for profit, but to meet the mutual obligations of kinship and co-residence. Aware that the government held different views about the cause of the disease, they examined their own ways of thinking about the world, but an alternative was literally ‘unthinkable’. The epidemic provided sorry evidence that the cultural restraints on killing by sorcery, like the limits placed on killing enemies in warfare, had been disregarded. The days passed and speakers had faced the problem from every direction. Angry men should kill just one man, destroy his dog or cut down his banana trees. One thing was enough. Kuru attacks were excessive.

In February 1963, a government census patrol came through the South Fore. Particular attention was given to providing an accurate count of kuru deaths in the past year and the names of any new cases. Following the census at Wanitabe, it soon became apparent that three new cases had gone unreported. In the preceding weeks, many people were aware that new cases had arisen, even as they pledged an end to sorcery. The threat to call upon the kiap to take the suspected sorcerers into exile was already being reconsidered and was never carried out.

The question of whether the epidemic was caused by sorcerers, so often addressed at the kuru meetings, would arise again in the 1990s as people reflected on the past. By then kuru was becoming rare, the political and social order had changed (Papua New Guinea had been an independent nation for more than 15 years) and discussions began to absorb new information, new experiences and to present additional views about the cause of kuru and its demise. As the great debates of the 1960s and the more informal discussions in the 1990s show, the Fore quest for truth is at the heart of sorcery beliefs, which seek to assign cause for severe illness, misfortune and death by identifying the persons responsible. This socio-medical analysis of the epidemic does not rest on germ theory. Nevertheless, it should not be viewed as a mere metaphor or fiction.

Fore narratives about the history of kuru are told in two forms: one as a story about the sequential purchase of sorcery knowledge and technology from Uwami in the north to Purosa in the south, and the other as a story we would identify more readily as history. In the latter, the Fore spoke of their first encounters with the new disease and provided the names of the victims. The first could be said to be indigenous social epidemiology, providing the rationale for the second. Together, they tell the same history.

As the kuru epidemic draws to a close, kuru sorcerers are no longer believed to occupy the Fore landscape, and a few Fore even speak of kuru as a form of sickness, but they have not changed their views about sorcery in general. Sorcery still provides an explanation for other severe ailments and misfortunes, a belief inscribed in the Sorcery Act of 1971, now part of the Revised Laws of the Independent State of Papua New Guinea, which defines acts of sorcery to be illegal. Nor do the Fore assume, as many scholars have mistakenly supposed, that all belief in sorcery and witchcraft would disappear with ‘modernization’ and modern science. This has not been the case in Papua New Guinea or elsewhere in the world. The Fore are correct, however, in assuming that ‘modernity’ had a place in ridding them of kuru. As a result of their encounters with those early messengers of modernity, the missionaries and colonial administrators who spoke out against cannibalism, which they considered to be a perversion and a legal offence, the Fore gradually stopped consuming deceased relatives. The Fore, the missionaries and the government officers saw no relationship between kuru and consuming the dead, but all had contributed in their own way to halting the transmission of the disease. Anthropologists and medical investigators did not bring an end to the epidemic. Our scientific understanding of the way in which kuru was transmitted and the nature of the infectious agent, however, results from the joint endeavours of anthropology and medicine.

The Fore have contributed most to this chapter of medical history. Victims of the disease, they provided a reliable history of their encounter with kuru, gave blood samples and the bodies of ailing and deceased relatives for scientific analysis and continue to work as research assistants for the study of prion-related disorders.
